# Comparative Evaluation of Deep Versus Superficial Erector Spinae Plane Blocks for Postoperative Analgesia in Modified Radical Mastectomy

**DOI:** 10.7759/cureus.113607

**Published:** 2026-07-29

**Authors:** Kuber J Bhinde, Lalit Gupta, Bharti Wadhwa, Sukhyanti Kerai

**Affiliations:** 1 Department of Anaesthesiology, Maulana Azad Medical College and Lok Nayak Hospital, New Delhi, IND

**Keywords:** analgesia, breast neoplasms, modified radical mastectomy, nerve block, pain

## Abstract

Background and aims

The erector spinae plane (ESP) block is a popular technique for postoperative analgesia across various surgical procedures. This study aimed to compare two different approaches - the superficial ESP (SES) block and deep ESP (DES) block - for the duration and quality of analgesia in women undergoing modified radical mastectomy (MRM).

Materials and methods

This prospective, double-blind, randomized comparative study was conducted on 44 American Society of Anesthesiologists (ASA) physical status I/II patients aged 18-65 years and posted for MRM. Patients were randomly assigned to either Group DES or Group SES. Following induction of anesthesia, patients were turned lateral, and the ultrasound-guided blocks were performed at the level of the fourth thoracic vertebra (T4) with 20 mL of 0.25% bupivacaine and 6 mg dexamethasone, as per group allocation. Time to first request of supplemental analgesia, postoperative numeric rating scale (NRS) scores, and total analgesic consumption over 24 hours postoperatively were recorded.

Results

There was no significant difference in time to first request for supplemental analgesia between the two groups. Group DES demonstrated a lower percentage of patients requesting supplemental analgesia compared to Group SES (18.2% vs. 63.6%, p = 0.002). Additionally, Group DES had significantly lower total postoperative analgesic consumption (17.05 ± 39.63 mg vs. 51.14 ± 42.59 mg of diclofenac, p = 0.004) and reduced NRS scores at 12 hours (0.55 ± 1.224 vs. 1.86 ± 1.885, p = 0.01) as compared to Group SES.

Conclusion

The ESP block is an effective regional anesthetic technique for the provision of postoperative analgesia in patients undergoing MRM. The DES block provided better quality of analgesia compared with the SES block.

## Introduction

Breast cancer is the most common malignancy among women worldwide. It accounted for an estimated 2.3 million new cases globally in 2020, representing 11.7% of all cancer cases. In India, breast cancer accounted for 13.5% of all cancer cases and 10.6% of all deaths [1].

Modified radical mastectomy (MRM) involves removal of the complete breast, including the tumor, overlying skin, and axillary lymphatics. Perioperative analgesia provision is of immense importance; 30%-50% of patients report moderate to severe acute pain postmastectomy, and 8%-25% report persistent postsurgical pain [2-4].

Various nerve and myofascial blocks have been described for pain relief following breast surgery; these include the thoracic paravertebral block (TPVB) [5], the PECS I and II blocks [6], the serratus anterior plane block, and thoracic epidural analgesia.

The erector spinae plane (ESP) block, first described in 2016, has two approaches - the deep ESP (DES) block and the superficial ESP (SES) block [7]. There is limited evidence comparing the DES and SES blocks for MRM in terms of time to first request of rescue analgesia. We hypothesized that the DES block would provide better postoperative analgesia than the SES block after MRM, by virtue of the proximity of drug deposition in the immediate vicinity of the dorsal and ventral rami of spinal nerves. The primary objective was to compare and evaluate the duration of analgesia provided by both blocks. The secondary objective was comparison and assessment of the total amount of analgesics required by the patients over 24 hours postoperatively.

## Materials and methods

This prospective, double-blind, randomized comparative study was conducted at a tertiary care hospital in Delhi after obtaining approval from the Institutional Ethics Committee (Approval Number: F.1/IEC/MAMC/MD/MS(96/02/2023/No. 86); dated May 15, 2023) and registration with the Clinical Trials Registry of India (Registration Number: CTRI/2023/09/058003 dated September 26, 2023). The study was conducted in accordance with the principles of the Declaration of Helsinki, 2013, and Good Clinical Practice guidelines. Written informed consent was obtained from all patients for participation in the study and for use of study data for research and educational purposes.

We enrolled 44 female patients aged 18-65 years with American Society of Anesthesiologists (ASA) physical status I and II who underwent MRM under general anesthesia (GA). Patients with body mass index (BMI) greater than 35 kg/m², a history of neuropsychiatric disorders, local site infections, coagulopathies, thrombocytopenia, pregnancy, and those on chronic treatment with opioids were excluded from the study.

**Figure 1:**
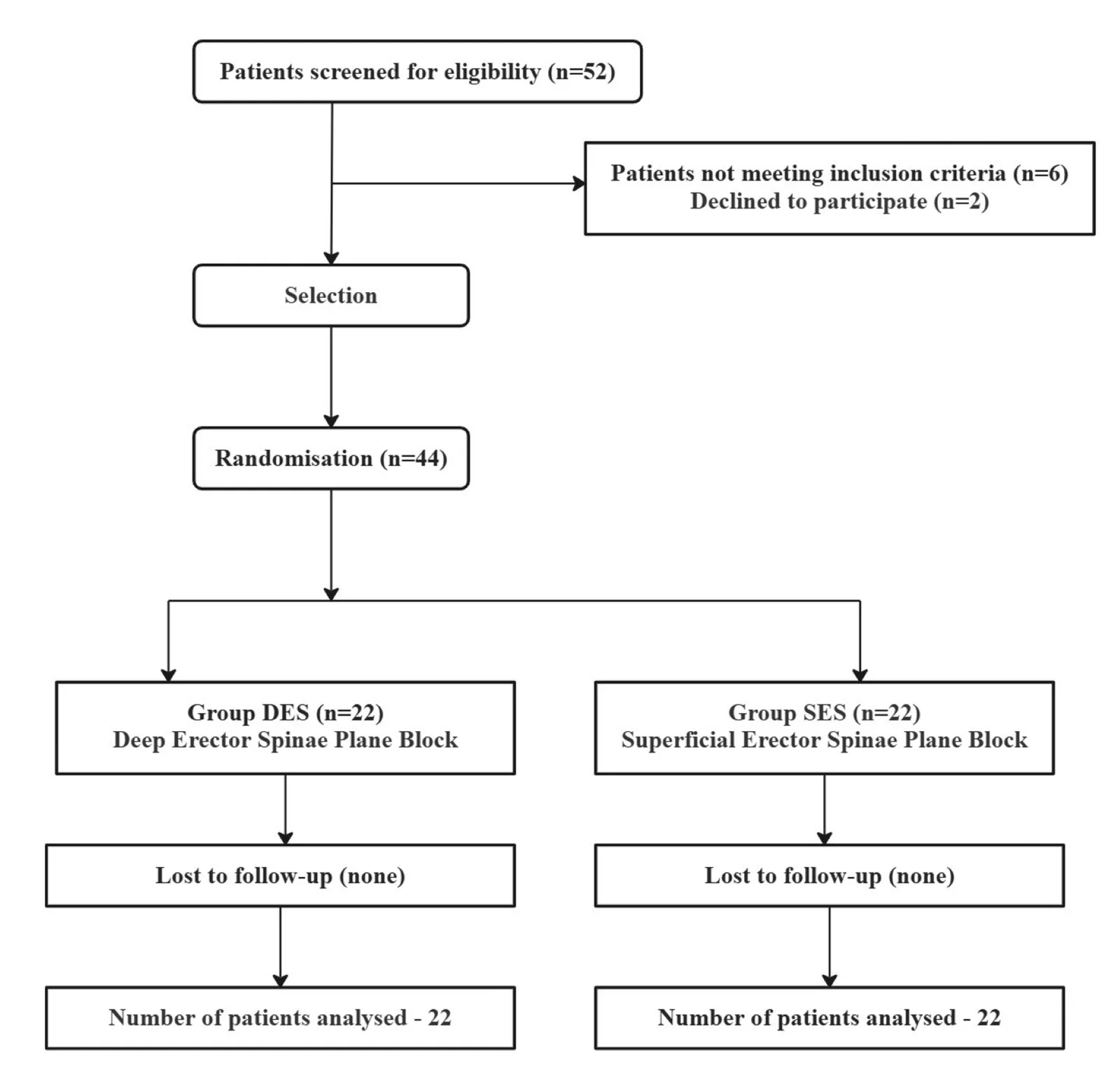
CONSORT flow diagram

Patients scheduled to undergo elective MRM were screened during pre-anesthetic evaluation, and eligible patients were enrolled for participation in the study (Figure 1). Patients were randomized into two groups - Group DES and Group SES - using computer-generated random number tables. The allocation of patients was concealed in sequentially numbered, sealed, opaque white envelopes.

To ensure blinding, the patient and the investigator following up the patient in the postoperative period were unaware of the group allocation (the block was administered to patients after induction of GA). The anesthesiologist following up the patient postoperatively would not administer the block to the patient. The block was administered by an experienced anesthesiologist who had performed more than 30 ultrasound-guided ESP blocks.

All patients were informed (in the language they understood) regarding the procedure that would be performed, and written informed consent was obtained. The patients were familiarized with the use of the numeric rating scale (NRS), identifying 0 as no pain and 10 as the worst imaginable pain [8].

On arrival in the operation theatre, standard anesthesia monitors, including continuous electrocardiogram (ECG), non-invasive blood pressure (NIBP), and pulse oximetry, were attached, and baseline values were noted. An intravenous (IV) access was secured on the non-operative forearm of the patient, and infusion of lactated Ringer’s solution was started.

Intravenous fentanyl 2 µg/kg was administered for analgesia. GA was induced with administration of IV propofol 1.5-2.5 mg/kg and IV rocuronium 0.6 mg/kg to facilitate muscle relaxation. The airway was secured with an appropriate device (Proseal® Laryngeal Mask Airway (Teleflex Inc., Wayne, PA, USA), size 3 or 4 as per the weight band of the patient), and anesthesia was maintained with a gaseous mixture of isoflurane in oxygen and nitrous oxide (50:50). Isoflurane administration was titrated by monitoring depth of anesthesia using Conox® (Fresenius Kabi, Bad Homburg, Germany) monitor, with a target qCON value between 40 and 60.

**Figure 2:**
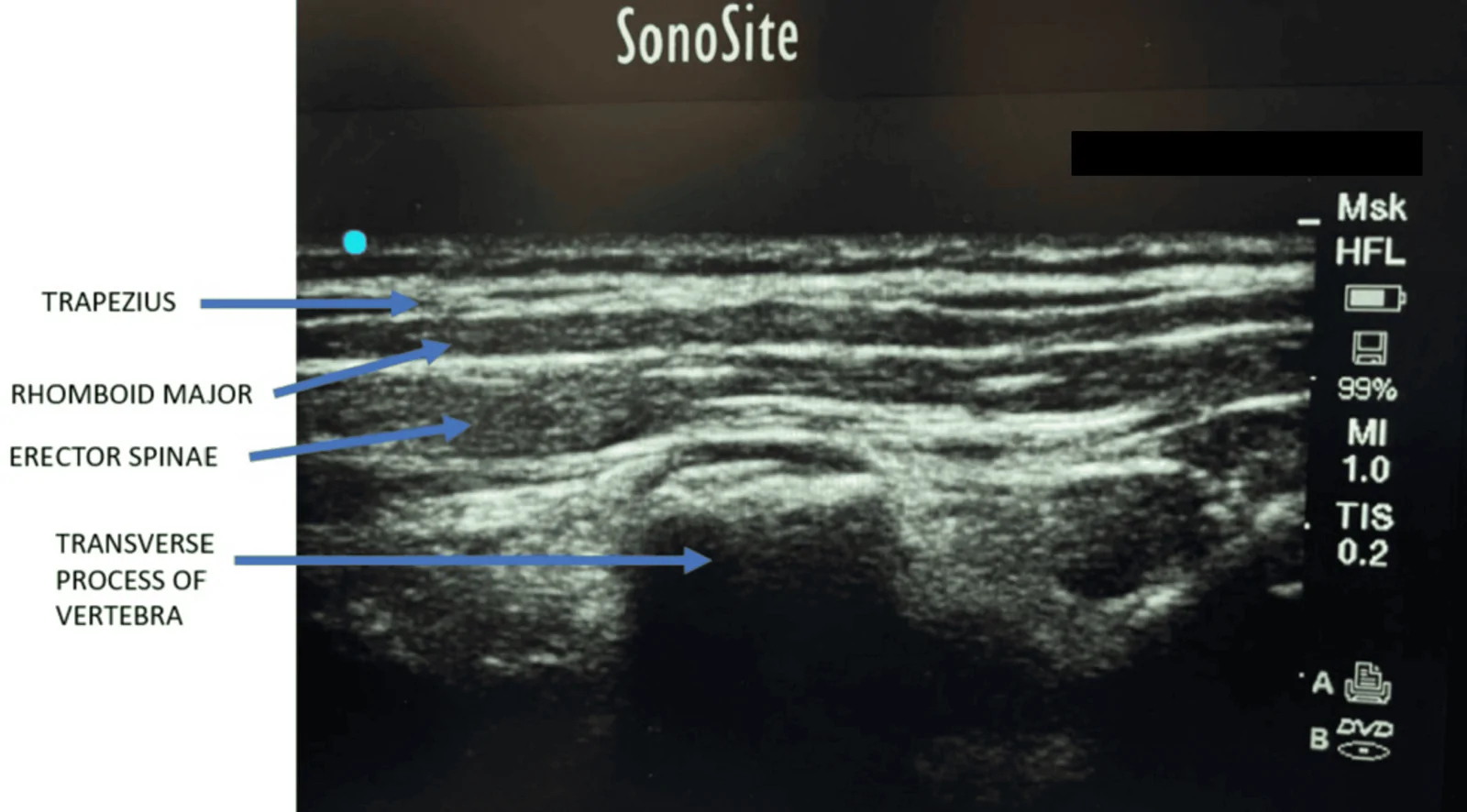
Ultrasound anatomy showing the transverse process and three muscle layers in the thoracic regions

The patient was then placed in a lateral position to perform the ESP block as per the group allocation. After cleaning, draping, and palpating for the T3 spinous process adjacent to the root of the scapular spine, an ultrasound machine (SonoSite®) was arranged, and a covered, sterile ultrasound probe was used to identify the transverse process of the T4 vertebra on the operative site in the sagittal plane. The ESP block in the study groups was administered using a 100 mm, 21-gauge echogenic cannula needle (PAJUNK® SonoPlex, Geisingen, Germany), inserted in-plane in the cephalad-caudal direction (Figure 2). In Group DES, the drug was administered deep to the erector spinae muscle plane and above the tip of the transverse process. The drug was administered above the erector spinae muscle (in the plane between the erector spinae and the rhomboid major) in Group SES. The drug administered for the block was 20 mL of 0.25% bupivacaine with 6 mg dexamethasone. The superior and inferior spread of the solution was visualized to confirm drug deposition in the correct plane according to the allocated group.

CONOX® (Fresenius Kabi, Bad Homburg, Germany) monitoring was used to guide additional IV fentanyl bolus requirements in doses of 0.5 µg/kg when the qNOX value rose above 60. qNOX was used to guide the titration of intraoperative analgesics, as it is more objective and specific than conventional responses and hemodynamic changes [9].

Hemodynamic changes were monitored using continuous ECG, pulse oximetry, and NIBP. All patients received IV ondansetron 4 mg prior to skin closure, and neuromuscular blockade was antagonized with IV neostigmine 50 µg/kg and IV glycopyrrolate 10 µg/kg. The airway device was removed, and patients were shifted to the postoperative room for monitoring.

Postoperative pain was assessed using the NRS on shifting the patient to the postanesthesia care unit (PACU) (considered as 0 minutes) and at 30 minutes and 2, 4, 6, 12, and 24 hours after surgery. For postoperative analgesia, patients in both study groups received IV paracetamol 1 g every eight hours. If the patient complained of pain with an NRS score greater than 3, IV diclofenac 1.5 mg/kg was administered as the rescue analgesic. The time to the first request for rescue analgesia was noted. If pain was still not relieved 30 minutes after administration of IV diclofenac, a second analgesic (IV tramadol 2 mg/kg) was administered. If, at any of the study intervals, the patient was comfortably sleeping, they were considered pain-free, with an NRS score of 0. The total analgesic consumption over the first 24 postoperative hours was recorded in both groups. Patient satisfaction was assessed using the seven-point Likert patient satisfaction score after 24 hours [10]. Any side effects (e.g., hematoma formation and pneumothorax) were noted.

The sample size for this study was estimated based on comparing the mean time to the first request for supplemental analgesia (minutes) between the two groups; we defined a clinically relevant minimum difference of 90 minutes in the time to the first request for supplemental analgesia between the two groups, based on the study by Sagar et al. [11]. Thus, a sample size of 21 patients per group, with an effect size of 1.0, provided 90% power to detect a significant difference between the two groups at an alpha level of 0.05. We included 22 patients per group in the study to account for attrition.

Statistical analysis was performed with IBM SPSS Statistics for Windows, Version 25.0 (Released 2017; IBM Corp., Armonk, NY, USA). Continuous variables were presented as mean ± SD, and categorical variables were presented as absolute numbers and percentages. Normally distributed continuous variables were compared using the unpaired t-test, whereas the Mann-Whitney U test was used for variables that were not normally distributed. Categorical variables were analyzed using either the chi-square test or Fisher’s exact test. A p-value less than 0.05 was considered statistically significant.

## Results

This prospective, randomized, comparative study was conducted over one year, from October 2023 to October 2024. A total of 52 patients posted for MRM were screened for eligibility, and 44 patients were enrolled for participation in the study. All enrolled patients received the intended study intervention and completed the study (Figure 1).

The demographic characteristics of patients in the study groups were comparable. There was no significant difference in the total intraoperative fentanyl requirement or the surgical duration between the study groups (Table 1).

**Table 1:** Demographic characteristics, intraoperative IV fentanyl requirements, and duration of surgery (n = 44)

Parameter	Group DES (n = 22) (mean ± SD)	Group SES (n = 22) (mean ± SD)	p-value (unpaired t-test)	Test statistic value (degrees of freedom)
Age (in years)	50.18 ± 9.027	49.36 ± 8.415	0.757	0.311 (42)
Weight (in kg)	61.05 ± 11.62	61.27 ± 11.01	0.947	-0.067 (42)
Height (in meter)	1.58 ± 0.07	1.58 ± 0.06	0.809	-0.243 (42)
BMI (kg/m^2^)	24.52 ± 4.03	24.45 ± 3.75	0.955	0.057 (42)
Intra-operative IV fentanyl requirements (µg)	9.55 ± 15.268	5.91 ± 11.406	0.376	0.895 (42)
Duration of surgery (minutes)	162.27 ± 39.992	154.77 ± 41.504	0.545	0.610 (42)

The mean time to first rescue analgesia was 9.88 ± 5.14 hours in the DES group and 10.43 ± 4.35 hours in the SES group. The mean difference was -0.55 hours (95% CI, -3.45 to 2.34), with no statistically significant difference between the groups (p = 0.721).

Four patients (18.2%) in the DES group and 14 patients (63.6%) in the SES group required rescue analgesia. The relative risk of requiring rescue analgesia in the DES group was 0.29 (95% CI, 0.11 to 0.73), indicating a significantly lower requirement than in the SES group (p = 0.002).

Mean postoperative diclofenac consumption was significantly lower in the DES group than in the SES group (mean difference, -34.09 mg; 95% CI, -59.12 to -9.06; p = 0.004).

Postoperative satisfaction scores were significantly higher in the DES group (mean difference, 0.46; 95% CI, 0.04 to 0.88; p = 0.022) (Table 2).

**Table 2:** Postoperative parameters recorded Data are presented as mean ± SD or number (percentage), as appropriate; MD = mean difference (DES - SES). RR: relative risk; CI: confidence interval; NRS: numeric rating scale; SD: standard deviation; DES: deep erector spinae plane block; SES: superficial erector spinae plane block

Outcome	Group DES (n = 22)	Group SES (n = 22)	Effect estimate (95% CI)	p-value	Statistical test
Patients requiring rescue analgesia, n (%)	4 (18.2)	14 (63.6)	RR = 0.29 (0.11 to 0.73)	0.002	Chi-square test
Time to first rescue analgesia (hours), mean ± SD	9.88 ± 5.14	10.43 ± 4.35	MD = -0.55 (-3.45 to 2.34)	0.721	Mann-Whitney U test
NRS score at 12 hours, mean ± SD	0.55 ± 1.22	1.86 ± 1.89	MD = -1.31 (-2.28 to -0.34)	0.010	Mann-Whitney U test
Diclofenac consumption during first 24 hours (mg), mean ± SD	17.05 ± 39.63	51.14 ± 42.59	MD = -34.09 (-59.12 to -9.06)	0.004	Mann-Whitney U test
Patient satisfaction score (7-point Likert scale), mean ± SD	5.41 ± 0.80	4.95 ± 0.58	MD = 0.46 (0.04 to 0.88)	0.022	Mann-Whitney U test

The NRS scores of the patients were comparable between the groups at the time of PACU admission (0 minutes) and at 30 minutes and 2, 4, 6, and 24 hours postoperatively. At 12 hours postoperatively, patients in the DES group had significantly lower NRS scores than those in the SES group (mean difference, -1.31; 95% CI, -2.28 to -0.34; p = 0.010) (Table 3).

**Table 3:** NRS scores at various time intervals in the study groups U refers to the test statistic value in the Mann-Whitney U test. NRS: numeric rating scale; DES: deep erector spinae plane block; SES: superficial erector spinae plane block

NRS	DES group (n = mean ± SD)	SES group (n = mean ±SD)	p-value (Mann-Whitney U Test)	Test statistic value
NRS-0 hours	0.86 ± 0.834	0.95 ± 1.090	0.940	U = 239
NRS-30 minutes	0.86 ± 0.710	1.05 ± 1.253	0.980	U = 241
NRS-2 hours	1.23 ± 0.922	1.55 ± 1.101	0.315	U = 201
NRS-4 hours	1.32 ± 1.129	1.50 ± 1.012	0.328	U = 202.5
NRS-6 hours	1.77 ± 1.020	2.23 ± 1.378	0.281	U = 197.5
NRS-12 hours	0.55 ± 1.224	1.86 ± 1.885	0.010	U = 147.5
NRS-24 hours	1.00 ± 0.816	1.18 ± 0.795	0.416	U = 210

No block-related complications were observed in either of the study groups.

## Discussion

In this study, we found that the duration of postoperative analgesia provided by ESP block is comparable, whether the local anesthetic drug is injected in the plane deep below or superficial to the erector spinae muscle. The time to first request for rescue analgesics in our study was 9.87 ± 5.13 hours in the DES group compared with 10.42 ± 4.34 hours in the SES group (p = 0.721). Studies on ESP block for mastectomies have reported durations of postoperative analgesia ranging from 5 hours to 41 hours [12-14]. The duration of action of a single-shot ESP block is dependent on the type and volume of local anesthetic chosen, the adjuvant used, and the site of injection. Malawat et al. found that postoperative analgesia with ESP block using 25 mL of 0.5% bupivacaine with 8 mg of dexamethasone in patients undergoing MRM lasted for approximately 41 hours [12]. On the other hand, Sinha et al. reported the duration of analgesia with ESP block (using 20 mL of 0.2% ropivacaine) to be five hours [13]. In our study, we used 20 mL of 0.25% bupivacaine with 6 mg of dexamethasone for ESP block in both groups. These results are similar to those reported by Thiagarajan et al.; they used the same volume of 0.25% bupivacaine for ESP block (i.e., 20 mL), while dexamethasone was given intravenously [14]. As expected, dexamethasone was found to increase the duration of analgesia, as seen in the studies by Malawat et al. and Thiagarajan et al. [12,14].

Although the primary outcome of time to first rescue analgesia was comparable between the two groups, the DES block demonstrated advantages across several clinically relevant secondary outcomes, suggesting improved overall quality of postoperative analgesia.

In Group DES, fewer patients requested rescue analgesia compared with Group SES (18.2% in Group DES vs 63.6% in Group SES; p = 0.002). Patients in Group DES had lower NRS scores at 12 hours postoperatively, a lesser requirement for postoperative rescue analgesics, and greater patient satisfaction compared with Group SES (Table 2). Therefore, the quality of analgesia provided by the DES block was superior to that provided by the SES block. In the DES group, the drug was deposited closer to the dorsal and ventral rami of the spinal nerves; in this plane, the cranio-caudal spread of the drug was likely to be greater and, hence, covered multiple dermatomes [7].

In the DES group, the mean IV diclofenac consumption was significantly lower compared with the SES group (Table 2). These observations align with those made by Sinha et al. In their study, postoperative analgesic requirements were significantly lower in patients who received the DES block [15]. However, the rescue analgesic used was morphine, whereas we used diclofenac in our study.

Sinha et al. found that injection of the drug deep to the ESP muscle leads to greater dermatomal coverage of the posterolateral chest wall compared with injection superficial to the muscle, as confirmed by pin-prick testing [15]. Cutaneous sensory testing for assessing the success of neural blockade in regional anesthesia has been suggested by some authors [15,16]; however, anatomical variations in the anastomotic interconnections between nerves have been described, leading to inconsistent patterns of cutaneous blockade with ESP block [17]. A cadaveric study on the spread of injectate following ESP block suggested that dye deposited deep to the erector spinae muscle stains both the dorsal and ventral rami as it gradually diffuses to the paravertebral and epidural spaces [18]. Injection of dye superficial to the erector spinae muscle resulted in staining only of the dorsal rami [7]. This could explain the superior analgesic efficacy of the DES block, as demonstrated in our study.

In our study, no patient in either group required IV tramadol postoperatively (second-line rescue analgesic) for analgesia. This avoided the deleterious side effects of opioids. The opioid-sparing effect of the ESP block has also been demonstrated in a study by Gürkan et al. [19].

In a retrospective analysis by Exadaktylos et al., patients who had undergone breast cancer surgery were analyzed for metastasis and cancer recurrence. The risk of cancer recurrence was significantly lower in patients receiving analgesia by a regional technique (paravertebral block) compared with patients receiving postoperative morphine analgesia [20]. Regional anesthesia has also been shown to attenuate the surgical stress response, which reduces immunosuppression, thereby preserving the immune system's capacity to eliminate residual cancer cells [21]. The goal of our study also aligns with the aim of reducing perioperative opioid requirements and elucidating the role of opioid-free anesthesia in cancer surgeries.

Our study adds to the literature by filling a gap in the current knowledge about pain management strategies in MRM. Few studies address the ideal plane of injection of local anesthetic while performing the ESP block. In fact, our study shows that both the DES and SES blocks can have a significant opioid-sparing effect. Findings from this research can help anesthesiologists make evidence-based decisions regarding the choice of analgesic technique and the optimal plane of injection of local anesthetic while performing the ESP block, thereby improving patient outcomes and satisfaction.

In our study, we have used qNOX to guide intraoperative analgesic supplementation. qNOX has been shown to rationalize analgesic consumption by better titration of analgesic drugs [9]. Both groups had similar IV fentanyl requirements intraoperatively (Table 1).

We acknowledge that there are certain limitations to the present study. It was a single-center study conducted on a small number of patients. The duration of analgesia was assessed by the time to request the first rescue analgesic, which depends on many patient-related factors. Sleeping patients without behavioral or hemodynamic indicators of pain were recorded as having no clinically significant pain at that assessment. We acknowledge that this may underestimate pain intensity. Larger sample sizes and multicentric trials are needed to build on our preliminary findings and further elaborate on the applications of DES and SES blocks for postoperative analgesia. Future studies can consider administering the block prior to induction of GA to allow sensory mapping of the block.

## Conclusions

This study demonstrates that both DES and SES blocks are effective regional anesthetic techniques for postoperative analgesia following MRM. Although the primary outcome, time to first rescue analgesia, was comparable between the two groups, the DES block was associated with superior secondary outcomes, including fewer patients requiring rescue analgesia, lower postoperative diclofenac consumption, lower pain scores at 12 hours, and higher patient satisfaction. These findings suggest that the DES block may provide better overall quality of postoperative analgesia.
